# Jumping Translocations of 1q in Myelodysplastic Syndrome and Acute Myeloid Leukemia: Report of Three Cases and Review of Literature

**DOI:** 10.1155/2018/8296478

**Published:** 2018-09-09

**Authors:** T. Couture, K. Amato, A. DiAdamo, P. Li

**Affiliations:** ^1^Clinical Cytogenetics Laboratory, Department of Genetics, Yale School of Medicine, New Haven, CT, USA; ^2^Diagnostic Genetics Sciences Program, Department of Allied Health Sciences, University of Connecticut, Storrs, CT, USA

## Abstract

Jumping translocations of 1q refer to the break-off of chromosome 1q as a donor fusing to two or more recipient chromosomes. We detected jumping translocations of 1q in three patients with initial diagnosis of myelodysplastic syndrome (MDS) and later progression to acute myeloid leukemia (AML). Review of literature found jumping translocations of 1q in 30 reported cases of MDS and AML. The cytogenetic findings from these 33 cases showed that seven cases had a stemline clone and 26 cases had de novo jumping translocations of 1q in which 5% of cell lineages had additional structural rearrangements. In 75% of cases, the 1q donor jumped to the short arm of recipient acrocentric chromosomes. Approximately 82% of the fusions occurred in the telomeric regions of short and long arms and 18% occurred in the pericentric or interstitial regions of recipient chromosomes. Hypomethylation of the donor 1q pericentromeric region and shortened telomeres in recipient chromosomes were associated with the formation of jumping translocations. Jumping translocations of 1q as an indication of chromosomal instability pose high risk for progression of MDS to AML and a poor prognosis. Further understanding of underlying genomic defects and their clinical significance will improve overall treatment and patient care.

## 1. Introduction

Jumping translocations are a rare type of cytogenetic abnormality initially detected in a case of acute monocytic leukemia and later found in various types of leukemias [1–3]. Jumping translocations occur when one donor chromosome segment breaks off and fuses to two or more recipient chromosomes in a successive manner and result in clonal abnormalities with a few cell lineages sharing a gain of the donor chromosome segment. The fusions of the break-off donor chromosome segment to telomeric or interstitial regions of recipient chromosomes form different chromosomal patterns of jumping translocations from different patients and among different types of leukemias [1–3]. The most commonly seen jumping translocations involving a 1q as the donor chromosome segment are referred to as jumping translocations of 1q. In this report, we present cytogenetic findings of jumping translocations of 1q from three cases initially diagnosed with myelodysplastic syndrome (MDS) and later progressed to acute myeloid leukemia (AML) and the additional 30 cases from review of the literature [1–10]. These results provide more information of the chromosomal patterns, underlying formation mechanisms, and clinical significance of jumping translocations of 1q in MDS and AML.

## 2. Case Presentation and Review of the Literature

A retrospective review of cases with MDS and AML from the CytoAccess database in Yale Cytogenetics Laboratory found three cases with jumping translocations of 1q [11]. Chromosome and fluorescence in situ hybridization (FISH) analyses were performed following laboratory's standardized protocols. Review of literature specific for jumping translocations of 1q in MDS and AML found 30 cases from ten reports [1–10]. Two cases with jumping translocations involving other donor chromosomes of 11q and 13q12 [7] and one case with jumping translocations of 3q13.1 were excluded [10].

### 2.1. Case 1

This male patient was first evaluated at age 59 with an indication of leukopenia and mild anemia; cytogenetic analysis found a normal result. Two years later, this patient was diagnosed with MDS and chromosomal analysis on a bone marrow aspirate showed an abnormal clone with jumping translocations of 1q. The karyotype was as follows: 46,XY,der(15)t(1;15)(q12;p12)[7]/46,XY,der(21)t(1;21)(q12;p12)[3]/46,XY,dup(1)(q21q42)[2]/46,XY,der(14)t(1;14)(q12;p12)[2]/46,XY[6]. FISH analysis using dual color probes for the PBX1 gene at 1q23.3 and TCF3 gene at 19p13.3 detected three copies of 1q in 32% of bone marrow cells. This patient started chemotherapy and then received a bone marrow transplant in two months. Six months later, the patient progressed to AML and died at age 62. Final chromosomal analysis revealed a karyotype of 46,XY,der(1;15)(q12;p12),del(20)(q11.2q13.3)[15]. FISH tests confirmed three copies of 1q and a deletion at 20q in 83.5%-96.5% of bone marrow cells.

### 2.2. Case 2

This patient, a 63-year old male, was referred with MDS by an indication of MDS with excess blasts. Chromosomal analysis showed a karyotype of 46,XY,der(15)t(1;15)(q12;p11.2)[4]/46,XY,der(22)t(1;22)(q12;p11.2)[2]/46,XY[7]. FISH analysis using dual color probes for the CDKN2C gene at 1p32.3 and CKS1B gene at 1q21.3 confirmed three copies of 1q in 5.5% of bone marrow cells. Over the next six months, the percentage of abnormal cells increased despite two rounds of chemotherapy and the patient progressed to AML. A transit clone with the der(15)t(1;15) and a deletion at 11q was noted. The last chromosome analysis revealed a karyotype of 46,XY,der(15)t(1;15)(q12;p11.2)[13]/46,XY,der(22)t(1;22)(q12;p11.2)[2]. FISH detected three copies of 1q in 94% of bone marrow cells. This patient died eight months after the initial finding of the jumping translocations of 1q.

### 2.3. Case 3

This patient was a male at age 73 when diagnosed with MDS. Chromosomal analysis showed a karyotype of 46,XY,der(15)t(1;15)(q12;p11.2)[13]/46,XY[7]. FISH confirmed three copies of 1q in 82.5% of bone marrow cells. Two years later, the patient progressed to AML and died soon after the last chromosome analysis which revealed a karyotype of 46,XY,der(15)t(1;15)(q12;p11.2)[9]/46,XY,der(22)t(1;22)(q12;p11.2)[4]/46,X,der(Y)t(Y;1)(q12;q11),t(10;17)(p10;p10)[3]. FISH detected three copies of 1q in 94% of bone marrow cells.

**Figure 1:**
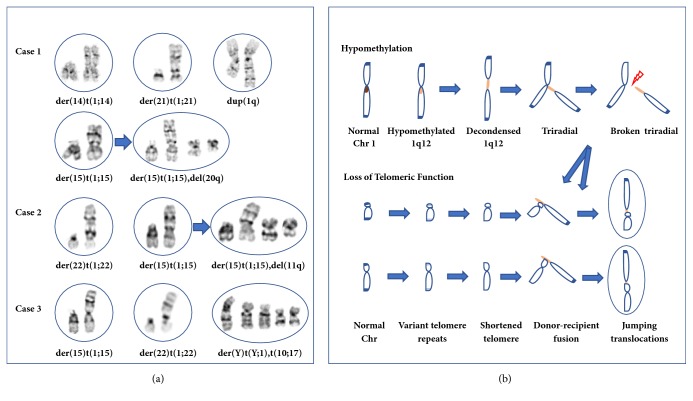
(a) Jumping translocations of 1q observed in the three patients. The jumping translocation in each cell lineage is shown inside a circle. Arrow points to an additional rearrangement evolved in a cell lineage. (b) Epigenetic alterations in the formation of jumping translocations of 1q. Hypomethylation of 1q12 pericentric heterochromatin induced chromatin decondensation, triradial formation, and break-off of 1q donor segment. Loss of telomeric function by variant telomeric repeats and shortened telomeres produced instable recipient chromosomes. The successive fusions between break-off donor segment and recipient chromosomes resulted in clonal abnormalities with cell linages sharing gains of donor chromosome segment.

**Figure 2:**
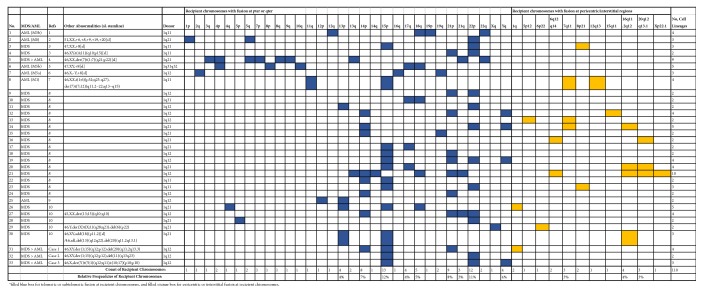
Patterns of donor and recipient chromosomes in jumping translocations of 1q of MDS and AML patients.

The cytogenetic findings of successive studies from these three patients all showed persistent jumping translocations of 1q with an increased percentage in bone marrow cells. The chromosome patterns of jumping translocations of 1q are shown in Figure 1(a). Review of literature specific for jumping translocations of 1q in MDS and AML found 30 cases from ten reports [1–10]. The clinical indications and patterns of jumping translocation of 1q from these 33 cases are summarized in Figure 2. For a total of 110 cell lineages from these 33 cases, the cell lineages with jumping translocations of 1q in a patient ranged from two to ten, with an average of about three cell lineages. Stemline abnormalities before the emerging of jumping translocations of 1q were noted in seven cases (No. 2-7, 30), seemingly unrelated clones were noted in two cases (No. 8 and 27), related clones with a jumping translocation and an additional rearrangement were noted in two cases (No. 29 and 33), an evolved abnormal clone with an additional aberration of a deletion in a 20q was noted in one case (No. 31), and a transit abnormal clone with an additional aberration of a deletion in an 11q was noted in one case (No. 32). These results indicated that at least 39% (13/33) of cases with jumping translocations of 1q had other clonal abnormalities. However, in cases with de novo jumping translocations of 1q, other structural rearrangements are rare events and only occur in 15% (4/26) of cases and in about 5% (4/79) of cell lineages with jumping translocations of 1q. The donor chromosome of 1q showed breakpoints in the pericentric region of 1q11, q12, and q21 except for one case with the breakpoint at 1q31q32. In terms of recipient chromosomes, 82% of the fusions occurred most likely in the telomeric regions of short (p) and long (q) arms and 18% occurred in the pericentric or interstitial regions of a chromosome. The most frequently seen telomeric fusions were 15p (12%), 22p (11%), 21p (8%), 14p (7%), 13p (4%), Yq (4%), 17q (4%), 18q (4%), and 21q (3%). The most frequently seen pericentric or interstitial fusions were 16q11.2q12 (4%), 7q11 (3%), and 20q12q13.1 (3%). Jumping translocations of 1q fusing with the short-arm telomeric regions of at least one of the acrocentric chromosomes 13, 14, 15, 21, and 22 were noted in 75% (25/33) of cases.

## 3. Discussion

The results from these 33 cases revealed the chromosomal fusion patterns between the 1q donor chromosome and different recipient chromosomes. However, all studies done by routine chromosome analysis lacked the molecular characterization of breakpoints involving the chromosome fusion, underlying genomic imbalances, and tumor gene mutations. A recent study using genomic array testing and targeted gene panel next-generation sequencing detected the 100–107 Mb duplication of 1q with breakpoints in 1q21.1-q21.3 region and recurrent mutations in the TET2 and SF3B1 genes [10]. The TET2 gene encodes a methylcytosine dioxygenase that catalyzes the conversion of methylcytosine to 5-hydroxymethylcytosine. Mutations in this gene have been associated with MDS (OMIM#612839) and responsiveness to hypomethylating agents such as azacitidine or decitabine [10]. The SF3B1 gene encodes subunit 1 of the splicing factor 3b protein complex. Splicing factor 3b, together with splicing factor 3a and a 12S RNA unit, forms the U2 small nuclear ribonucleoproteins complex (U2 snRNP). Mutations in the SF3B1 gene have been seen in MDS (OMIM#614286). These findings supported further genomic analysis to define the breakpoints involving the jumping translocations and disease-related gene mutations [12, 13].

Approximately one-third of MDS/AML cases with jumping translocations of 1q either emerged from a stemline abnormal clone or evolved additional clonal abnormalities and two-thirds were detected as de novo chromosomal abnormalities. Shortened telomeres and many variant telomeric repeats were detected in the fusion points from jumping translocations of 1q in a case of MDS progressed to AML; this result suggested that the extended proliferation of cancer cells might be related to the loss of telomeric function [4]. Copy number gain of 1q involving pericentromeric breakpoints had been seen frequently in hepatocellular carcinoma. It was postulated that hypomethylation of satellite 2 DNA at 1q12 alters the interaction between the CpG-rich satellite DNA and chromatin proteins which results in heterochromatin decondensation, breakage, and aberrant 1q formation [14]. Investigation of der(16)t(1;16)/der(1;16) in breast carcinomas suggested that hypomethylation of pericentromeric satellite 2 DNA in 1q12 might be associated with the accumulation of many numerical chromosome alterations and aggressive histological features [15]. The study of five patients with 1q12 rearrangements causing multiple myeloma observed that hypomethylation of the 1q12 pericentromeric heterochromatin induced transient decondensations and triradials of the 1q12 pericentromeric region. Triradials of 1q12 originated clonal unbalanced 1q rearrangements or jumping translocations by successive fusing to the telomeric or pericentromeric regions of recipient chromosomes [16]. Therefore, the presence of jumping translocations indicated chromosomal instability by altered epigenetic modifications. A proposed mechanism for the formation of jumping translocations is schemed in Figure 1(b).

The progression of MDS to AML and poor prognosis were noted in the present three cases with jumping translocations of 1q. This result was consistent with previous findings in which jumping translocations of 1q were associated with a high risk of progression to AML and poor prognosis and thus warranted aggressive therapy [8, 10]. Currently, the azanucleosides azacitidine and decitabine are used for the treatment of MDS and AML [17]. These azanucleosides are referred to as hypomethylating agents which interfere with the DNA methylation machinery. The incorporation of azacytidine into RNA or decitabine into DNA triggers two main antitumor cellular activities through the induction of cytotoxicity leading to DNA damage response and DNA hypomethylation through inhibition of DNA methyltransferase which enables reactivation of tumor suppressor genes for normal growth and differentiation. For MDS and AML with jumping translocations of 1q induced by hypomethylation at 1q pericentric heterochromatin, the treatment by azanucleosides may rely on the cytotoxicity effect. Further studies to understand the underlying genomic defects causing jumping translocations and mechanism of action for targeted treatment are needed.

In summary, jumping translocations of 1q have been shown as an indication of chromosomal instability likely induced by epigenetic alterations of hypomethylation and shortened telomeres. The jumping translocations of 1q showed unique rearrangement patterns with multiple cell lineages which all have a gain of donor chromosome segment and lack other complex structural or numerical rearrangements. It is not clear whether these epigenetic alterations occur independently as a two-step event or concurrently by driving genetic defects. Further molecular analyses to understand the genetic defects causing jumping translocations of 1q and related disease-causing mechanism can facilitate targeted therapy for this type of MDS and AML.
